# Thrombotic Thrombocytopenic Purpura: A Tale of Two Cases

**DOI:** 10.7759/cureus.21853

**Published:** 2022-02-02

**Authors:** Prakash R Ghogale, Ashutosh Kumar Pandey, Edavan Pulikkanath Praveen, Prabhakar Yadav, Saurabh Pathak

**Affiliations:** 1 Nephrology, SSPM Medical College and Lifetime Hospital, Sindhudurg, IND; 2 Anaesthesiology, SSPM Medical College and Lifetime Hospital, Sindhudurg, IND; 3 Biochemistry, SSPM Medical College and Lifetime Hospital, Sindhudurg, IND; 4 Nephrology, Tata Main Hospital, Jamshedpur, IND

**Keywords:** thrombotic thrombocytopenic purpura, red-cell fragmentation, microangiopathic haemolytic anaemia, steroids, plasmapheresis, ttp

## Abstract

Microangiopathic hemolytic anaemia, thrombocytopenia, renal failure, neurologic abnormalities, and fever form the pentad of thrombotic thrombocytopenic purpura (TTP). Early diagnosis is crucial because TTP responds well to plasmapheresis therapy but is associated with substantial mortality if left untreated. A substantial percentage of patients with TTP used to die from systemic microvascular thrombosis in the brain and the heart. However, since plasma exchange therapy became a mainstay in the treatment of TTP, mortality has reduced considerably. Diagnosing TTP can be difficult due to the vast range of symptoms and the absence of clearly defined diagnostic criteria. Hemolytic uremic syndrome and disseminated intravascular coagulation are a close differential of TTP. Here we report two patients with TTP who achieved remission when treated with steroids, plasmapheresis and were free of disease relapse till about two months during follow-up in the outpatient department.

## Introduction

Thrombotic thrombocytopenic purpura (TTP) is one of the entities in thrombotic microangiopathy (TMA). Eli Moschcowitz first defined TTP in 1924, and it is characterised by a pentad of symptoms, including microangiopathic haemolytic anaemia, thrombocytopenia, renal failure, neurologic abnormalities, and fever. TTP affects around 4-6 million people per year [1]. TTP has a 90% death rate if left untreated. The mortality rate is lowered to 10-20% with plasma exchange therapy. Another entity in TMA syndrome is haemolytic-uremic syndrome (HUS), which is defined by the triad of microangiopathic haemolytic anaemia, microvascular occlusion, and thrombocytopenia [1]; in the past, they were considered one disease due to the comparable clinical features [2]. TTP is also linked to fever and neurological symptoms, however, TTP and HUS are clinically indistinguishable. The appearance of neurological abnormalities or acute kidney injury, respectively, raises clinical suspicion of TTP or HUS. Disseminated intravascular coagulation (DIC) is also one of the differential diagnoses [3]. Proof of steroid efficacy in the treatment of TTP is still low. Here we report two cases of TTP admitted to a rural hospital. Their clinical and blood profiles, disease course, follow up after hospitalization and treatment modalities are described. The efficacy in sustaining the remission of TTP by the use of corticosteroids, once plasmapheresis is stopped and also at the follow up is being described. The other aim is to provide insight into TTP management.

## Case presentation

Patient 1

A 38-year-old man presented to our hospital with chief complaints of fever for six days and altered sensorium for two days. He was treated elsewhere for the past four days and then shifted to our hospital. On examination he was febrile - 104^o^F, haemodynamically stable, and general examination revealed no abnormality, Glasgow coma scale (GCS) was E4M5V3. He was evaluated accordingly, his reports done were: blood urea- 63.9 mg/dL, serum creatinine- 1.97 mg/dL, sodium- 141 mEq/L, potassium- 3.22 mEq/L, chloride- 103.2 mEq/L, hemoglobin- 14.6 g/dL, platelet count- 42000/cumm, tropical fever workup came negative, serology was negative, blood and urine cultures were sterile, Chest X-ray (CXR) was normal, procalcitonin (PCT) was 50 ng/ml, prothrombin time (PT) and activated partial thromboplastin time (aPTT) were normal, direct and indirect Coombs test was negative, urine routine; protein +++, glucose ++, pus cell 3-/HPF, red blood cell (RBC) 25-30/HPF, ultrasound abdomen- right kidney (RK)- 11.7 x 5.8cm, left kidney (LK)- 10.7 x 6.1 cm, raised bilateral echogenicity, cortico-medullary differentiation (CMD) maintained. Liver function test (LFT)- bilirubin (direct/Indirect) 1.17/0.9 mg/dL, aspartate transaminase (AST or SGOT) and alanine transaminase (ALT or SGPT) were normal, HbA1c was 9.2%. MRI Brain showed no significant brain parenchymal abnormality. After sending cultures, the patient was empirically started on intravenous antibiotics: injection piperacillin tazobactam + teicoplanin, injection artesunate with other supportive treatment. On day 1 evening, he became violent with the inability to control by restraint, was sedated and intubated, in view of the prolonged need of sedation and risk of respiratory depression, put on a mechanical ventilator. Lactate dehydrogenase (LDH) was 90 U/L on day 1. Repeat LDH on day 2 was 589 U/L which increased to 987 U/L on day 3. On day 3, renal function test (RFT) showed blood urea 191 mg/dl, creatinine 5.15 mg/dl and since morning his urine output had decreased, so the patient was haemodialysed. Direct and indirect Coombs tests were negative and peripheral smear for schistocytes was reported as negative. In view of fever persisting over 102 - 106^o^F despite broad-spectrum antibiotics and antimalarial drugs, the elevation of LDH within three days, fall in hemoglobin, deranged RFTs, altered sensorium, and thrombocytopenia with normal coagulation parameters; a diagnosis of thrombotic thrombocytopenic purpura was made. His mean corpuscular volume (MCV) was 78.7 fl and the PLASMIC score was 6 points (high risk) denoting 72% risk of severe ADAMTS13 deficiency (defined as ADAMTS13 activity level <15%). Injection methylprednisolone 1 gm was started post hemodialysis on day 3. On day 4, LDH was 1443 U/L and the first session of plasmapheresis with an exchange of 2.5 litres followed by haemodialysis (HD) was done. The patient dramatically became afebrile after the first session of plasmapheresis, while prior to plasmapheresis he had a fever of 102 - 104^o^F. A second dose of injection methylprednisolone was given post HD. Improvement in LDH level and platelet count was seen on day 5 and the second session of plasmapheresis with an exchange of 2.5 litres of plasma and the third dose of injection methylprednisolone 1 gm was given. Improvement in LDH and platelet count was seen on day 6 and day 7 and the third and fourth session of plasmapheresis with an exchange of 2.5 litres was done and oral prednisolone 1 mg/kg was started on day 6. By day 8 his sensorium had improved, and he was oriented, he was extubated, and his blood reports were urea- 137 mg/dL, creatinine- 2.08 mg/dL, sodium- 139 mEq/L, potassium- 3.41 mEq/L, chloride- 98 mEq/L, hemoglobin- 11.3 g/dL, platelet count- 208000 per cumm, LDH- 705 U/L. In view of urine output of 2550 mL, plasmapheresis was withheld even though LDH was >500 U/L as his plasmapheresis filter was not reusable and it was decided to wait and see the trend of the platelet count and LDH to decide about the further need for plasmapheresis. His blood pressure started increasing, he needed Injection nitroglycerin infusion and later started on oral antihypertensive drugs. From day 9, serial RFT and platelet count showed improvement and no further plasmapheresis or hemodialysis was needed till discharge. He was shifted to ward on day 11 and after his blood sugar level was also controlled (initial days after methylprednisolone pulses he needed insulin infusion and then shifted on to regular insulin with oral hypoglycaemic drug). After it was ascertained that his RFT, platelet count, and LDH were stable he was discharged on day 14 on antihypertensive drugs, mixture insulin and oral hypoglycaemic, and prednisolone 1 mg/kg with a plan to continue the same dose for a total of one month and then taper and omit. His daily lab reports and events are shown in Table 1.

**Table 1:** Day-wise investigation chart of patient 1 *Plasmapheresis was done as follows; 2.5L of plasma exchange per cycle (50 mL/kg) blood flow 150 mL/min, heparin anticoagulation and 2.5L fresh frozen plasma as replacement fluid. LDH: Lactate dehydrogenase; HD: haemodialysis

Day of admission	Serum Urea (mg/dL)	Serum Creatinine (mg/dL)	Serum Sodium (mEq/L)	Serum Potassium (mEq/L)	Blood Hemoglobin (g/dL)	Total Leukocyte Count (per cumm)	Platelet Count (per cumm)	Serum LDH (U/L)	Daily Urine Output (mL)	Procedure
1	63.9	1.97	141	3.22	14.6	8700	42000	90	450	
2					12	10400	36000	589	950	
3	191	5.15	146	4.46	10.3	5000	30000	987	100	HD
5	220	5.02	147	4.75	11.9	8200	63000	1443	700	Plasmapheresis* + HD
6	205	3.39	149	4.42	10.4	6200	79000	929	2120	Plasmapheresis* + HD
7	166	2.81	152	3.17	11.1	10700	195000	586	2800	Plasmapheresis*
8	153	2.5	140	2.82	11.3	11600	223000	607	2400	Plasmapheresis*
9	137	2.08	139	3.41	11.3	9900	208000	705	2550	Extubated
10	140	2	137	4.37	9.8	9900	328000	498	2880	
11	122	1.89	141	3.08	9.7	11400	361000	558	>2500	Shifted to ward
12	99	1.7	141	3.36	10.5	19200	450000	656	>2500	
13	99	1.68	140	3.55				734	>2500	
14	72	1.57	140	3.72	10.8	16400	516000	669	>2500	Discharged
Follow-up day after discharge	On follow-up in OPD									
3	38	1.24	142	4.42	9.6	12400	343000	550		
8	36	1.14			10.4	13300	306000	444		
13	26	0.96			11.7	11700	237000	357		
21	31	0.85			12.1	17100	348000	395		
28	21	0.76			11.2	13000	322000	373		
53	16	0.66			11.2	10000	370000			

Patient 2

A 27-year-old man presented to our ICU with fever, alteration in sensorium, and decreased urine output. On examination and investigation, he was found to have fever, disorientation, thrombocytopenia, renal dysfunction, and microangiopathic haemolytic anaemia. A diagnosis of TTP was made. His mean corpuscular volume (MCV) was 86 fl and the PLASMIC score was 6 points (high risk) denoting 72% risk of severe ADAMTS13 deficiency (defined as ADAMTS13 activity level <15%). He was initiated on three pulses of injection methylprednisolone followed by oral prednisolone 1 mg/kg for one month and then taper and omit. Alternate day haemodialysis was done till his urine output improved and daily plasmapheresis was done till his platelet count increased above 150000 per cumm. Tropical fever workup investigation was negative, HIV, hepatitis B surface antigen (HBsAg), and hepatitis C virus (HCV) were also negative, blood and urine cultures were sterile, CXR was normal, PCT was 0.42 ng/mL, PT and aPTT were normal, direct and indirect Coombs test was negative. Urine routine showed protein +++, RBC >50/HPF, pus cells 4-5/HPF, urine protein creatinine ratio (UPCR) 2651 mg/g creatinine. Ultrasound abdomen showed RK- 11.7 x 5.8cm, LK- 10.7 x 6.1 cm, raised bilateral echogenicity and CMD maintained. LFT- bilirubin was normal. Serum SGOT (1082 U/L) and SGPT (220 U/L) were raised on admission but later normalized. HbA1c was 6%. His daily lab reports and events are shown in Table 2.

**Table 2:** Day-wise investigation chart of patient 2 *Plasmapheresis was done as follows: 3.5L of plasma exchange per cycle (50 mL/kg) blood flow 150 mL/min, heparin anticoagulation and 3.5L fresh frozen plasma as replacement fluid. LDH: Lactate dehydrogenase; HD: haemodialysis

Day of admission	Serum Urea (mg/dL)	Serum Creatinine (mg/dL)	Serum Sodium (mEq/L)	Serum Potassium (mEq/L)	Blood Hemoglobin (g/dL)	Total Leukocyte Count (per cumm)		Platelet Count (per cumm)			Serum LDH (U/L)		Daily Urine Output (mL)	Procedure
1	146	6.8	155	4.14	12.7	7900		42000			9485		100	HD
2	118	5	152	4.4	9.8	4200		27000					<50	Plasmapheresis*
3	176	8.4	148	4.8	9.6	5400		32000			2978		<50	Plasmapheresis* + HD
4	132	4.82	146	4.4	11.4	6800		57000			1645		<50	Plasmapheresis*
5					9.3	6800		58000			669		<50	Plasmapheresis* + HD
6	249	3.2	144	4.5	9.2	6000		60000			857		50	Plasmapheresis*
7	163	5.4	147	4.1	8.9	7600		105000			769		50	Plasmapheresis* +HD
8	72	2.8	146	3.3	8.1	8100		81000			877		40	Plasmapheresis*
9	232	7.25	148	3.7	7.7	8800		150000			891		190	Plasmapheresis* +HD
10	144	4.3	145	4	6.8	10200		150000			837		150	Plasmapheresis*
11	109	3.72	143	3.5	5.9	9900		165000			1002		230	Plasmapheresis* +HD
12					8.4	13800		211000			1075		1055	
13	221	8.4	139	3.9	7.1	11900		202000			1055			HD
14			138	4.2	8.6	11600		195000			1056		1400	
15	207	8.56	138	4.2	8.4	12200		193000					1500	HD
16	112	4.76	141	4	9	11800		246000					1350	
17	177	7.61	141	3.08	9.5	14000		186000			920		1950	HD
18	85	3.45												Discharged
Follow up Day After Discharge	On follow-up in OPD													
2	171	4.85			8.2	12300	196000		720	2400				
5	142	3								3100		HD canula removed		
10	49	1.27	136	4.2	9.7	16100	176000							
19	38	1.27	130	2.9	10.2	12500	183000							
26	28	1.15	128	3	10.4	11000	282000							

## Discussion

In adults, thrombotic thrombocytopenic purpura is idiopathic in around a third of instances, meaning it develops suddenly and without any known underlying cause. Women are more likely to be affected. The median age at the time of diagnosis is around 40 years old [4]. About two-thirds of cases of TTP are found in a variety of clinical situations, triggering an acute episode. Bacterial or viral infections, pregnancy (especially during the last trimester and the postpartum period), autoimmune disorders (mainly systemic lupus erythematosus and antiphospholipid syndrome) [5], disseminated malignancy, and bone marrow transplantation and ingestion of drugs could trigger TTP. The drugs may mediate TTP through acute immune-mediated toxicity (quinine, ticlopidine, clopidogrel) [6], insidious dose-related toxicity (mitomycin C, alpha-interferon, cyclosporine, tacrolimus). Other immunosuppressive and chemotherapeutic agents may also trigger TTP. These should be considered as an additional criterion to suspect TTP.

Laboratory data should be obtained in patients who come with new thrombocytopenia, with or without evidence of renal insufficiency and other aspects of classic TTP, to rule out DIC and evaluate for signs of microangiopathic hemolytic anemia. Increased lactate dehydrogenase and indirect bilirubin, decreased haptoglobin, and increased reticulocyte count, along with a negative direct antiglobulin test, all support the TTP diagnosis. Schistocytes should be looked for in the peripheral smear. The diagnosis of TTP in both cases was made on clinical grounds. According to the newer classification, both the cases fit mostly into primary TMA acquired - TTP due to ADAMTS13 (a disintegrin and metalloproteinase with a thrombospondin type 1 motif, member 13) antibodies triggered due to infection, as the elevated procalcitonin levels suggested in both cases.

Because plasmapheresis eliminates von Willebrand factor multimers, ADAMTS13 anti-metalloproteinase antibodies [3], and endothelial cytokines, plasma exchange remains the basis of TTP treatment [4]. Fresh frozen plasma (FFP) transfusion recovers ADAMTS13 deficiency [3]. Plasma exchange is continued for at least two days post the platelet count is normal and indications of haemolysis have disappeared. Breckenridge et al. used steroids alone, steroids with an antiplatelet drug, and plasmapheresis to treat 10 patients [7]. He came to the conclusion that the plasmapheresis arm had a higher survival rate. Glucocorticoids did not improve survival rates against plasmapheresis, according to William et al. [8]. Given the autoimmune nature of acquired TTP, there is a legitimate justification for the use of steroids in its treatment. The level of proof for steroid efficacy in the treatment of TTP, however, is still rather low. The use of glucocorticoids appears to be a viable method, despite the fact that it has never been examined in a big clinical trial. Other immunomodulatory treatments, such as rituximab, vincristine, cyclophosphamide, and splenectomy, have also been shown to be effective in refractory or relapsing TTP. Relapses are prevalent as well. 

The literature does not provide a treatment time, however, therapy should be continued until the platelet count reaches 100,000/mm^3 ^and the LDH levels fall below 400 U/l, as these are the most sensitive markers for assessing therapeutic response [9]. In both of our cases, platelet counts increased to above 150,000/mm^3^ for two days after which plasmapheresis was halted, although LDH levels showed an increasing trend immediately after plasmapheresis was stopped and later showed a downward trend. Thus corticosteroid therapy plays a role in maintaining complete disease remission, in both of our cases following the initial rise in LDH seen after halting plasmapheresis, later on, there was a downward trend in LDH. Outpatient follow-up is essential to monitor remission or detect recurrent chronic form with a mortality rate of roughly 15% [10] and we have follow-up data of two months in the first patient and one month in the second patient, both patients stayed in remission. Both our cases show that the addition of corticosteroid therapy helps in maintaining complete disease remission when plasmapheresis is stopped and also at follow-up. The drawback in our case report is that in both the cases the diagnosis was made on clinical grounds and the ADAMTS13 activity assay, ADAMTS13 functional inhibitor assay and anti-ADAMTS13 antibody assay and complement factor gene assay and antibodies were not done for availability and financial reasons. Our hospital and patients were from a rural setup. This also highlights the limitation in some country areas where patients are unable to bear the financial burden of expensive testing.

## Conclusions

Primary TTP is triggered by conditions such as pregnancy, infections, cancers, HIV, lupus, surgical stress, chemotherapy, or medications such as clopidogrel and ticlopidine. TTP is a serious condition and a keen eye is to be kept for diagnosing it when conditions which trigger it are present. Plasmapheresis is the cornerstone of therapy and has improved patient outcome considerably, and addition of corticosteroid therapy helps in maintaining complete disease remission when plasmapheresis is stopped and also at follow-up.
